# Supplementary material for: Patterns of Intron Gain and Loss in Fungi
Source: PLoS Biol. 2004 Nov 30;2(12):e422. doi: 10.1371/journal.pbio.0020422 (PMC532390; doi:10.1371/journal.pbio.0020422)
Supplement: Table S1 — Also available at http://genes.mit.edu/NielsenEtAl/. (4.3 MB ZIP). [file pbio.0020422.st001.zip › NielsenEtAl/html/190.html]

AN4064.1.NCU09477.1.MG06656.1.FG06021.1


```
 CLUSTAL W (1.82) Multiple Sequence Alignments - Introns Inserted


Sequence 1: NCU09477.1	313 aa
Sequence 2: FG06021.1	311 aa
Sequence 3: MG06656.1	306 aa
Sequence 4: AN4064.1	311 aa
Alignment Length: 315 aa
Number Identitical Residues: 236 aa
Alignment Score (without introns) 9822


MG06656.1 	---------MGMP0PFVVDFLM1GGVSAAVSKTAAAPIERVKLLVQNQ0EEMIRAGRLDR
NCU09477.1	--MAEQQKVLGMP0PFVADFLM~GGVSAAVSKTAAAPIERIKLLVQNQ0DEMIRAGRLDR
FG06021.1 	MSSAEPQKVLGMP0PFVADFLM~GGVSAAVSKTAAAPIERVKLLIQNQ~DEMLKTGRLDR
AN4064.1  	MASQSDKSVLGMP0GFVVDFLM1GGVSAAVSKTAAAPIERVKLLIQTQ~DEMLRSGRLDR
          	 :  . .. :***  **.**** *****************:***:*.* :**:::*****

MG06656.1 	RYDGIVDCFKRTSQAEGVMSLWRGNTANVIRYFPTQALNFAFRDKFKAMFGYKKDKDGYA
NCU09477.1	RYNGIIDCFKRTTADEGVMALWRGNTANVIRYFPTQALNFAFRDKFKKMFGYKKDVDGYW
FG06021.1 	KYNGIGDCFKRTMADEGVMSLWRGNTANVIRYFPTQALNFAFRDKFKKMFGYKKDKDGYA
AN4064.1  	KYNGLMDCFRRTTQAEGVVSLWRGNTANVIRYFPTQALNFAFRDTYKSMFAYKKDRDGYA
          	:*:*: ***:**   ***::************************.:* **.**** *** 

MG06656.1 	KWMAGNLASGG0AAGATSLLFVYSLDYARTRLANDAKSAKGGGDRQFNGLVDVYRKTLAS
NCU09477.1	KWMAGNLASGG~AAGATSLLFVYSLDYARTRLANDAKSAKKGGERQFNGLVDVYRKTIAS
FG06021.1 	MWMAGNLASGG~AAGATSLLFVYSLDYARTRLANDAKNAKSGGDRQFNGLVDVYKKTLAS
AN4064.1  	KWMAGNLASGG0MAGATSLLFVYSLDYARTRLANDAKSTKGGGERQFNGLIDVYKKTLAS
          	 **********  ************************.:* **:******:***:**:**

MG06656.1 	DGIAGLYRGFGPSVAGIVVYRGLYFGMYDSIKPVLLTGSLETNFFASFALGWCVTTGAGI
NCU09477.1	DGIAGLYRGFGPSVAGIVVYRGLYFGLYDSIKPVLLVGDLKNNFLASFALGWCVTTAAGI
FG06021.1 	DGIAGLYRGFMPSVAGIVVYRGLYFGMYDSIKPVVLTGNLQGNFLASFALGWIVTTGAGI
AN4064.1  	DGIVGLYRGFGPSVLGIVVYRGLYFGMYDSLKPVVLTGALEGSFLVSFLLGWGVTTGAGI
          	***.****** *** ***********:***:***:*.* *: .*:.** *** ***.***

MG06656.1 	ASYPLDTVRRRMMMTSGEAVKYSSSFDAFKQIVAKEGVKSLFRGAGANILRGVAGAGVLS
NCU09477.1	ASYPLDTIRRRMMMTSGEAVKYKSSFDAASQIVAKEGVKSLFKGAGANILRGVAGAGVLS
FG06021.1 	ASYPLDTIRRRMMMTSGEAVKYKNTMDAARQIVAKEGVKSLFKGAGANILRGVAGAGVLS
AN4064.1  	ASYPLDTVRRRMMMTSGEAVKYSSSFDAFRQIIAREGVKSLFKGAGANILRGVAGAGVLS
          	*******:**************..::**  **:*:*******:*****************

MG06656.1 	IYDQLQLIMFGKAFKGGSG
NCU09477.1	IYDQLQVLLFGKAFKGGSG
FG06021.1 	IYDQLQVLLFGKAFK----
AN4064.1  	IYDQVQLILFGKKFK----
          	****:*:::*** **
```
